# Transcription factor ATMIN facilitates chemoresistance in nasopharyngeal carcinoma

**DOI:** 10.1038/s41419-024-06496-x

**Published:** 2024-02-06

**Authors:** Xue-Liang Fang, Qing-Jie Li, Jia-Yi Lin, Cheng-Long Huang, Sheng-Yan Huang, Xi-Rong Tan, Shi-Wei He, Xun-Hua Zhu, Jun-Yan Li, Sha Gong, Han Qiao, Ying-Qin Li, Na Liu, Jun Ma, Yin Zhao, Ling-Long Tang

**Affiliations:** Department of Radiation Oncology, Sun Yat-sen University Cancer Center, State Key Laboratory of Oncology in South China, Collaborative Innovation Center for Cancer Medicine, Guangdong Key Laboratory of Nasopharyngeal Carcinoma Diagnosis and Therapy Center for Precision Medicine of Sun Yat-sen University, Guangzhou, 510060 PR China

**Keywords:** Cancer therapeutic resistance, Head and neck cancer

## Abstract

Despite that the docectaxel-cisplatin-5-fluorouracil (TPF) induction chemotherapy has greatly improved patients’ survival and became the first-line treatment for advanced nasopharyngeal carcinoma (NPC), not all patients could benefit from this therapy. The mechanism underlying the TPF chemoresistance remains unclear. Here, by analyzing gene-expression microarray data and survival of patients who received TPF chemotherapy, we identify transcription factor ATMIN as a chemoresistance gene in response to TPF chemotherapy in NPC. Mass spectrometry and Co-IP assays reveal that USP10 deubiquitinates and stabilizes ATMIN protein, resulting the high-ATMIN expression in NPC. Knockdown of ATMIN suppresses the cell proliferation and facilitates the docetaxel-sensitivity of NPC cells both in vitro and in vivo, while overexpression of ATMIN exerts the opposite effect. Mechanistically, ChIP-seq combined with RNA-seq analysis suggests that ATMIN is associated with the cell death signaling and identifies ten candidate target genes of ATMIN. We further confirm that ATMIN transcriptionally activates the downstream target gene LCK and stabilizes it to facilitate cell proliferation and docetaxel resistance. Taken together, our findings broaden the insight into the molecular mechanism of chemoresistance in NPC, and the USP10-ATMIN-LCK axis provides potential therapeutic targets for the management of NPC.

## Introduction

Nasopharyngeal carcinoma (NPC) is a malignant head and neck cancer with high occurrence in East and Southeast Asia, especially in Southern China [1]. Unfortunately, more than 70% of patients with NPC are classified as advanced disease at initial diagnosis [2]. For advanced NPC, chemoradiotherapy constitutes the backbone of treatment [3]. A randomized controlled phase III clinical trial has previously reported that docectaxel-cisplatin-5-fluorouracil (TPF) based-induction chemotherapy (IC) given before radiotherapy could improve survival of advanced NPC [4]. Consequently, TPF-based-IC is recommended as the first-line treatment for patients with advanced NPC [5]. However, tumors’ responses to TPF chemotherapy vary in clinical practice due to tumor heterogeneity [6], with ~10% of patients presenting with stable disease and having poor outcomes after receiving TPF chemotherapy [7]. Therefore, it is of urgent need to elucidate the underlying mechanism of TPF resistance for the development of potential therapeutic targets in NPC.

Transcription factors (TFs) are sequence-specific DNA-binding proteins that control gene transcription and gene expression by recognizing and binding cis-regulatory elements of target genes [8]. TFs are categorized into 73 families according to their DNA-binding domains, such as fork head domain, TEA domain, and zinc finger domain, and are involved in various biological processes [9, 10]. So far, around 20% of oncogenes have been reported as TFs and they play central roles in the development, metastasis and therapeutic response in cancers [11]. Previous studies have reported that transcription factor SNAIL drives tumorigenesis and metastasis in pancreatic cancer [12]; KLF5 maintains EMT and causes resistance to docetaxel in prostate cancer [13]; and HOXB9 confers cisplatin resistance in ovarian cancer [14]. In NPC, it has also been found that transcription factors such as HOPX, CBX1 and TEAD4 could promote metastasis and tumor growth [15–17]. Nevertheless, the biological function and the underlying mechanisms of TFs in regulating chemoresistance of NPC remain incompletely understood.

ATMIN, a TF belonging to the zinc finger protein family, has been reported to play a critical role in DNA damage repair, cell apoptosis, and organ morphogenesis [18–20]. Dysfunction of ATMIN also participates in cancer progression. Interestingly, ATMIN serves as either an oncogene or a tumor suppressor in a tissue-specific manner. For example, the activation of ATMIN/ATM pathway promotes the occurrence of glioblastoma [21], and ATMIN activates DYNLL1 to promote the progression of B cell lymphoma [22]; while ATMIN suppresses metastasis by altering the WNT-signaling pathway in colorectal cancer [23]. Also, ATMIN has low expression in lung adenocarcinoma and is associated with poor clinical outcomes [24]. Thus, the functional role of ATMIN in cancers remains elusive, especially its role in NPC progression has not been fully investigated.

In the present study, we revealed that transcription factor ATMIN is up-regulated and promotes docetaxel-resistance and tumor growth in NPC. Moreover, we identified USP10 as a potent deubiquitinase for ATMIN protein stabilization. We further confirmed that ATMIN is associated with the cell death signaling, and it transcriptionally activates LCK to facilitate the proliferation and chemoresistance of NPC cells. Overall, our findings indicate that USP10-ATMIN-LCK axis regulates NPC docetaxel sensitivity and promotes tumor growth, which may provide a potential therapeutic target for the management of NPC.

## Results

### ATMIN may serve as a chemoresistance gene in NPC patients

**Fig. 1:**
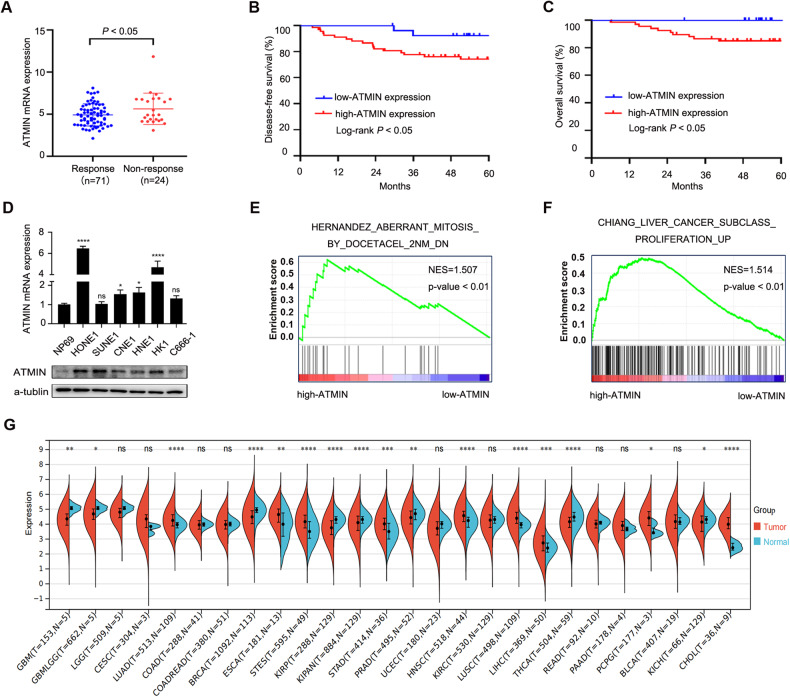
ATMIN expression is upregulated in chemoresistant patients and predicts poor prognosis. **A** ATMIN mRNA expression in NPC patients with response (*n* = 71) and non-response (*n* = 24) to TPF chemotherapy. Kaplan–Meier survival curves of disease-free survival (**B**) and overall survival (**C**) according to ATMIN mRNA expression in NPC patients receiving TPF chemotherapy. **D** ATMIN expression is up-regulated on mRNA (up) and protein level (down) in NPC cell lines. GSEA analysis based on RNA-seq results of 95 patients showing gene sets related to docetaxel response (**E**) and tumor proliferation (**F**). **G** Pan-cancer analysis of the mRNA expression of ATMIN in tumor and normal tissue samples with the TCGA dataset. Data in (**A**) are presented as mean ± SD, *P* values were calculated using Student’s *t* test (**A**), log-rank test (**B**, **C**), one-way ANOVA (**D**) or Wilcoxon rank sum test (**G**). *P < 0.05, **P < 0.01, ***P < 0.001, ****P < 0.0001. ns not significant. NPC nasopharyngeal carcinoma, GBM glioblastoma multiforme, GBMLGG glioma, LGG brain lower grade glioma, CESC cervical squamous cell carcinoma and endocervical adenocarcinoma, LUAD lung adenocarcinoma, COAD colon adenocarcinoma, COADREAD colon adenocarcinoma/rectum adenocarcinoma esophageal carcinoma, BRCA breast invasive carcinoma, ESCA esophageal carcinoma, STES stomach and esophageal carcinoma, KIRP kidney renal papillary cell carcinoma, KIPAN pan-kidney cohort (kidney chromophobe + kidney renal clear cell carcinoma + kidney renal papillary cell carcinoma), STAD stomach adenocarcinoma, PRAD prostate adenocarcinoma, UCEC uterine corpus endometrial carcinoma, HNSC head and neck squamous cell carcinoma, KIRC kidney renal clear cell carcinoma, LUSC lung squamous cell carcinoma, LIHC liver hepatocellular carcinoma, THCA thyroid carcinoma, READ rectum adenocarcinoma, PAAD pancreatic adenocarcinoma, PCPG pheochromocytoma and paraganglioma, BLCA bladder urothelial carcinoma, KICH kidney chromophobe, CHOL cholangiocarcinoma. The unprocessed images of the blots are shown in Supplementary Fig. S3.

Through analyzing our previous gene-expression microarray data GSE132112 [25], we found that the ATMIN expression was upregulated in NPC patients with non-response to TPF induction chemotherapy (Fig. 1A). Survival analysis revealed that higher ATMIN expression indicated shorter disease-free survival and overall survival in NPC patients who received TPF chemotherapy (Fig. 1B, C). Gene-expression profiles of NPC patients receiving TPF chemotherapy were also obtained from the Affiliated Hospital of Guilin Medical College. Consistently, a higher ATMIN expression was observed in the non-response group (Supplementary Fig. S1). Besides, both the mRNA and protein expression of ATMIN were up-regulated in NPC cell lines (HONE1, SUNE1, CNE1, HNE1, HK1 and C666-1) compared to that in normal nasopharyngeal epithelial cell line (NP69) (Fig. 1D). Gene set enrichment analysis (GSEA) found that the gene sets related to docetaxel resistance and proliferation were significantly enriched in high-ATMIN NPC samples using the GSE132112 dataset (Fig. 1E, F). To further explore the significance of ATMIN in cancers, we analyzed The Cancer Genome Atlas (TCGA) dataset and confirmed a general upregulation of ATMIN expression in solid tumors when compared to that in normal tissues (Fig. 1G). Taken together, these findings suggest that ATMIN may play a critical role in regulating chemoresistance of NPC.

### USP10 deubiquitinates and stabilizes the ATMIN protein

**Fig. 2:**
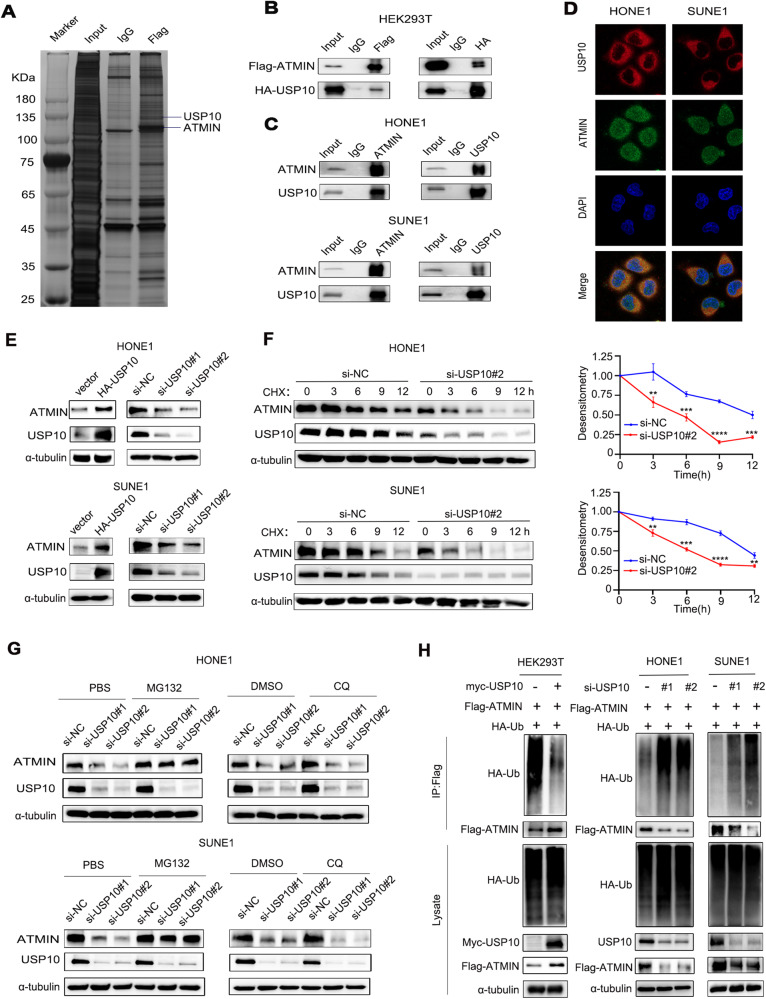
USP10 deubiquitinates and stabilizes the ATMIN protein. **A** Silver staining of FLAG-immunoprecipitated proteins separated from HONE1 cells overexpressing FLAG-ATMIN. Black lines indicated the proteins of interest. **B** Co-IP with anti-FLAG and anti-HA antibodies in HEK293T cells overexpressing FLAG-ATMIN and HA-USP10. **C** Co-IP with anti-ATMIN and anti-USP10 antibodies in HONE1 and SUNE1 cells. **D** Immunofluorescence staining revealed the cellular location of USP10 (red) and ATMIN (green) in HONE1 and SUNE1 cells. **E** Western blot analysis of ATMIN expression with USP10 overexpression or silencing in HONE1 and SUNE1 cells. **F** The effect of CHX treatment (left) and greyscale analysis of the results (right) in HONE1 and SUNE1 cells transfected with si-USP10#2 or si-NC. **G** The effect of MG132 (left) and CQ (right) treatment in HONE1 and SUNE1 cells transfected with indicated siRNA. **H** HEK293T cells (left) co-transfected with FLAG-ATMIN, HA-ubiquitin (Ub) and MYC-USP10 or the vector plasmids were subjected to Co-IP and immunoblotted with the indicated antibodies. HONE1 (middle) and SUNE1 (right) cells co-transfected with FLAG-ATMIN, HA-ubiquitin (Ub) and si-USP10#2 or si-NC were subjected to Co-IP and immunoblotted with the indicated antibodies. Data in (**F**) are presented as mean ± SD, *P* values were calculated using Student’s *t* test, **P < 0.01, ***P < 0.001, ****P < 0.0001. The unprocessed images of the blots are shown in Supplementary Fig. S3.

To explore the possible mechanism of the up-regulation of ATMIN expression in NPC, we overexpressed ATMIN in HONE1 cells, and performed mass spectrometry analysis to explore the potential ATMIN-binding proteins, and found the only deubiquitinase USP10 (Fig. 2A). The exogenous interactions between ATMIN and USP10 were confirmed in HEK293T cells by Co-IP assays (Fig. 2B), and the endogenous interactions between them were further confirmed in NPC cells (Fig. 2C). We then conducted the immunofluorescence staining assay to explore their potential locations in cells, and found that USP10 mainly located in the cytoplasm, and ATMIN located both in the cytoplasm and nuclear in NPC cells. What’s more, USP10 and ATMIN showed co-localization in the cytoplasm (Fig. 2D). Previous studies have reported that deubiquitinases (DUBs) could stabilize the protein expression by deubiquitinating the targeting proteins [26, 27], we thus further explore whether USP10 affects the protein expression of AMTIN. It was found that overexpressing USP10 increased the protein expression of ATMIN, while silencing USP10 exerted the opposite effect (Fig. 2E). Additionally, knockdown of USP10 promoted the degradation of ATMIN upon CHX treatment, as shown by the shorter half-life of ATMIN protein in the USP10-knockdown group (Fig. 2F).

To further confirm whether the accelerated degradation of ATMIN upon USP10-knockdown depends on the ubiquitin-proteasome pathway or the lysosomal pathway, NPC cells were treated with proteasome inhibitor MG132 or lysosome inhibitor CQ after USP10-knockdown. We found that the degradation of ATMIN upon USP10-knockdown could be reversed by MG132 rather than CQ (Fig. 2G), indicating that USP10 upregulates the ATMIN protein level through the ubiquitin-proteasome pathway. We next tested the effects of USP10 on the ubiquitination of ATMIN and observed that overexpressing USP10 inhibits the poly-ubiquitination of ATMIN, while silencing USP10 promotes the poly-ubiquitination of ATMIN (Fig. 2H). Altogether, these results reveal that USP10 deubiquitinates and stabilizes the ATMIN protein.

### Knockdown of ATMIN inhibits cell proliferation and facilitates docetaxel-sensitivity of NPC cells in vitro

**Fig. 3:**
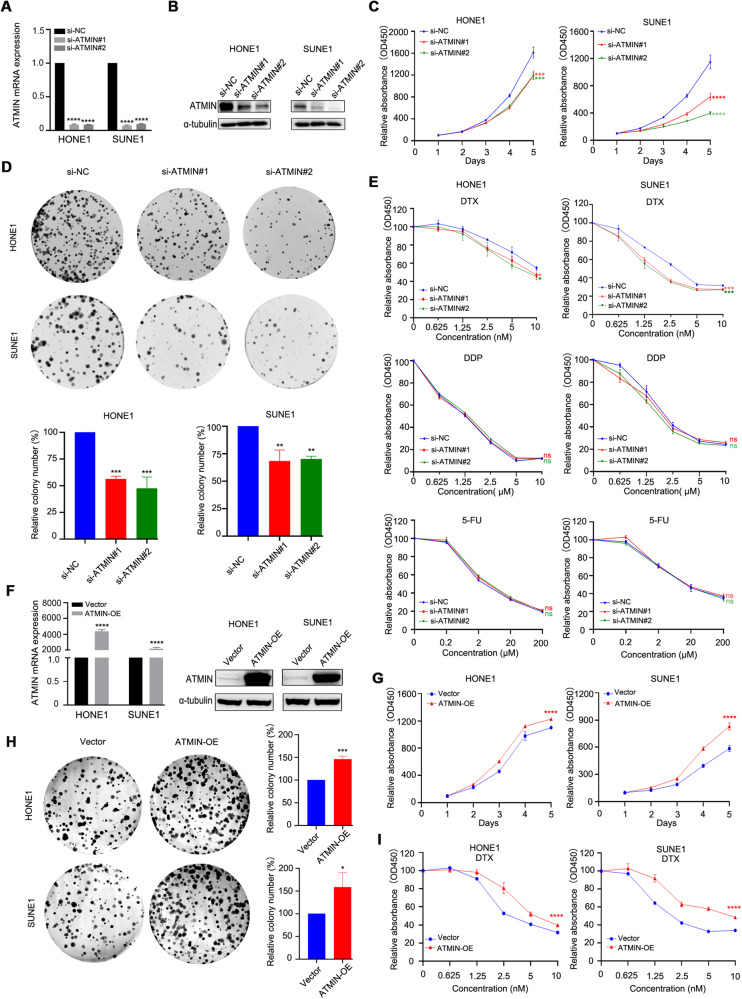
Knockdown of ATMIN inhibits cell proliferation and facilitates docetaxel-sensitivity of NPC cells in vitro. **A**, **B** qRT-PCR and western blot analysis of ATMIN expression in HONE1 and SUNE1 cells transfected with si-ATMIN#1 or si-ATMIN#2 or si-NC. **C** CCK-8 assays determining the growth curves of the transfected cells. **D** Representative images and quantification of the colony formation assays in the transfected cells. **E** CCK-8 assays testing the sensitivity of the transfected cells to the indicated doses of DTX, DDP and 5-FU. **F** qRT-PCR and western blot analysis of ATMIN expression in HONE1 and SUNE1 cells transfected with FLAG-ATMIN. **G** CCK-8 assays determining the growth curves of the SUNE1 and HONE1 cells transfected with vector or FLAG-ATMIN. **H** Representative images and quantification of the colony formation assays in the SUNE1 and HONE1 cells transfected with vector or FLAG-ATMIN. **I** CCK-8 assays testing the sensitivity of the SUNE1 and HONE1 cells transfected with vector or FLAG-ATMIN to the indicated doses of DTX. Data are presented as mean ± SD, *P* values were calculated using one-way ANOVA (**A**, **C**–**E**) or Student’s *t* test (**F**–**I**). *P < 0.05, **P < 0.01, ***P < 0.001, ****P < 0.0001. ns not significant. DTX docetaxel, DDP cis-diamminedichloro-platinum, 5-FU 5-fluorouracil. The unprocessed images of the blots are shown in Supplementary Fig. S3.

To explore the biological function of ATMIN in NPC, we knocked down ATMIN in NPC cells (Fig. 3A, B) and conducted cell viability assay and clonogenic assay, and found that ATMIN-knockdown constrained the proliferation of NPC cells (Fig. 3C, D). To confirm the function of ATMIN in TPF chemoresistance of NPC cells, we treated ATMIN-knockdown NPC cells with docetaxel, cisplatin or 5-fluorouracil, respectively, and performed CCK-8 assays. We observed that knockdown of ATMIN significantly increased the docetaxel sensitivity, but had no significant impact on cisplatin or 5-fluorouracil sensitivity (Fig. 3E). To further confirm the above results, we conducted cell viability assay, clonogenic assay and docetaxel-sensitivity assay in NPC cells overexpressed with ATMIN. It was found that overexpression of ATMIN could promote the proliferation and docetaxel-resistance of NPC cells (Fig. 3F–I), which further confirmed that ATMIN serves as a docetaxel-resistance gene in NPC.

### RNA-seq combined with ChIP-seq analysis suggests that ATMIN regulates cell death signaling

**Fig. 4:**
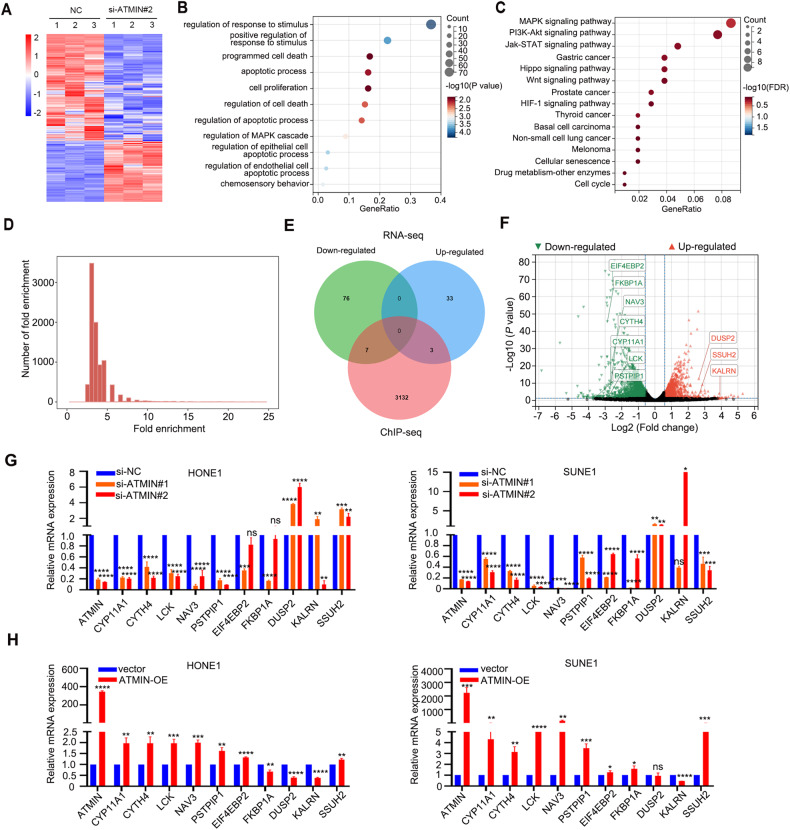
RNA-seq combined with ChIP-seq analysis suggests that ATMIN regulates cell death signaling. **A** RNA-seq and the heatmap analysis showed differentially expressed genes between HONE1 cells transfected with si-ATMIN#2 and si-NC. **B**, **C** GO and KEGG pathways enrichment analysis of RNA-seq results. **D** ChIP-seq analysis of peaks enriched by ATMIN in HONE1 cells with ATMIN overexpression (*q* value < 0.05). **E** Venn diagram showing 10 candidate genes between RNA-seq (|log_2_fold change| ≥ 2.5 and *q* value < 0.05) and ChIP-seq (*q* value < 0.05) peaks within 3000 bp of the transcription start sites. **F** Volcano plot showing the 10 candidate genes. **G**, **H** qRT-PCR showing the relative mRNA expression of 10 candidate genes with ATMIN silencing or overexpression in HONE1 and SUNE1 cells. Data are presented as mean ± SD, *P* values were calculated using one-way ANOVA (**G**) or Student’s *t* test (**H**). *P < 0.05, **P < 0.01, ***P < 0.001, ****P < 0.0001. ns not significant.

ATMIN is a transcription factor of the zinc finger family [20]. To dissect the mechanism underlying the oncogenic role of ATMIN in NPC, we performed RNA-seq after knocking down ATMIN and chromatin immunoprecipitation sequencing (ChIP-seq) with ATMIN overexpression in HONE1 cells to screen potential targets of ATMIN. The RNA-seq analysis identified 409 up-regulated genes and 744 down-regulated genes upon ATMIN-knockdown (Fig. 4A), including DYNLL1, which has been reported as a transcriptional target of ATMIN [22]. Gene Ontology (GO) analysis confirmed that these genes were enriched in response to stimulus, cell death, apoptosis, and proliferation process (Fig. 4B). These genes were also enriched in cancer-related signaling pathways such as MAPK, Jak-STAT, PI3K-Akt, Hippo, Wnt and HIF-1 signaling pathways through Kyoto Encyclopedia of Genes and Genomes (KEGG) analysis (Fig. 4C). Meanwhile, ChIP-seq analysis identified a total of 4332 peaks enriched with ATMIN overexpression (Fig. 4D). 10 candidate genes were obtained by combining the RNA-seq and ChIP-seq results, which includes 7 down-regulated genes and 3 up-regulated genes upon ATMIN-knockdown (Fig. 4E, F). These ten genes mainly function in regulating cancer progression according to previous reports [28–35]. To verify the regulation of ATMIN on these candidate genes, we overexpressed or knocked down ATMIN to examine the mRNA expression of these genes in NPC cells. It was found that knockdown of ATMIN suppressed the expression of CYP11A1, CYTH4, LCK, NAV3, PSTPIP1, EIF4EBP2, FKBP1A and promoted the expression of DUSP2 in both SUNE1 and HONE1 cells (Fig. 4G), while overexpression of ATMIN promoted the expression of CYP11A1, CYTH4, LCK, NAV3, PSTPIP1, EIF4EBP2, FKBP1A and inhibited the expression of DUSP2 and KALRN (Fig. 4H). The regulations on these ten genes mediated by ATMIN are mostly accordance with the RNA-seq results, indicating that ATMIN may regulate cell proliferation and chemoresistance through these genes.

### LCK functions as a downstream target gene of ATMIN in NPC cells

**Fig. 5:**
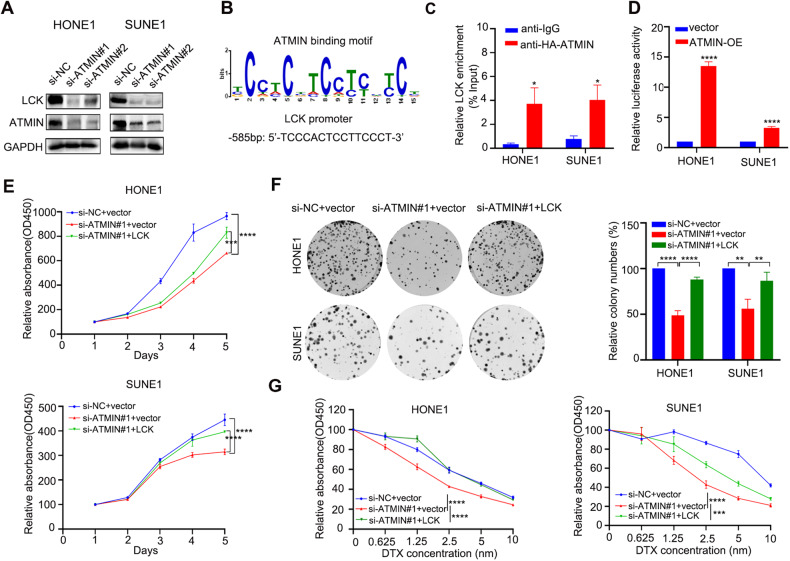
LCK functions as a downstream target gene of ATMIN in NPC cells. **A** Western blot analysis of LCK expression after ATMIN silencing. **B** ATMIN-binding motif and predicted LCK promoter site binding for ATMIN. **C** ChIP-qPCR validation of ATMIN enrichment on the promoter of *LCK*. **D** Dual-luciferase reporter assays in HONE1 and SUNE1 cells co-transfected with ATMIN plasmid or empty vector and pGL3-Basic-LCK plasmid (*n* = 3). **E** CCK-8 assays determining the growth curves of HONE1 and SUNE1 cells co-transfected with si-NC plus vector or si-ATMIN#1 plus vector or si-ATMIN#1 plus LCK plasmid. **F** Representative images and quantification of the colony formation assays in HONE1 and SUNE1 cells co-transfected with si-NC plus vector or si-ATMIN#1 plus vector or si-ATMIN#1 plus LCK plasmid. **G** CCK-8 assays in HONE1 and SUNE1 cells co-transfected with si-NC plus vector or si-ATMIN#1 plus vector or si-ATMIN#1 plus LCK plasmid followed with docetaxel treatment in an increased dose manner. Data are presented as mean ± SD, *P* values were calculated using Student’s *t* test (**C**, **D**) or one-way ANOVA (**E**–**G**). *P < 0.05, **P < 0.01, ***P < 0.001, ****P < 0.0001. The unprocessed images of the blots are shown in Supplementary Fig. S3.

To confirm the exact downstream target gene of ATMIN, we examined the correlations between the mRNA levels of ATMIN and these ten candidate genes in 113 NPC patients from the GSE102349 dataset, and found that the expression of LCK and FKBP1A, had prominent correlations with ATMIN expression (Supplementary Fig. S2). However, only the correlation between LCK and ATMIN is consistent with the RNA-seq and the qRT-PCR results after ATMIN-knockdown or ATMIN overexpression in NPC cells. We thus chose the LCK gene for further validation. In addition to that the mRNA expression of LCK was inhibited by ATMIN-knockdown, the protein expression of LCK was also inhibited in ATMIN-knockdown NPC cells (Fig. 5A). LCK is an oncogene which has been reported to promote oral cancer metastasis, glioma growth and ovarian tumor cisplatin resistance [36–38]. As ATMIN is a transcription factor that could directly activate gene transcription and expression, we thus looked for whether there are binding sites for ATMIN in the promoter of *LCK* gene. The binding motif of ATMIN was revealed by ChIP-seq analysis, and we found one binding site for ATMIN in the promoter of *LCK* gene (Fig. 5B). ChIP-qPCR assay confirmed that ATMIN could directly bind to the promoter of *LCK* gene (Fig. 5C). To further confirm that ATMIN drives the transcription of LCK, we cloned the upstream 1000 bp (starting from the transcription starting site of *LCK*) into the pGL3-Basic luciferase vector and performed the luciferase reporter assay. It was observed that ATMIN significantly enhanced the luciferase activity of *LCK* promoter (Fig. 4D). These findings confirmed that LCK is a downstream target gene of ATMIN in NPC cells.

To further validate whether LCK is a functional downstream target gene of ATMIN in NPC cells, we conducted cell viability assay, clonogenic assay and docetaxel-sensitivity assay, and found that overexpression of LCK could reverse the effects of ATMIN-knockdown-mediated cell proliferation suppression and docetaxel sensitization in NPC cells (Fig. 5E–G). Overall, these results showed that LCK is a functional downstream target gene of ATMIN in NPC.

### Knockdown of ATMIN suppresses tumor growth and enhances docetaxel-sensitivity of NPC in vivo

**Fig. 6:**
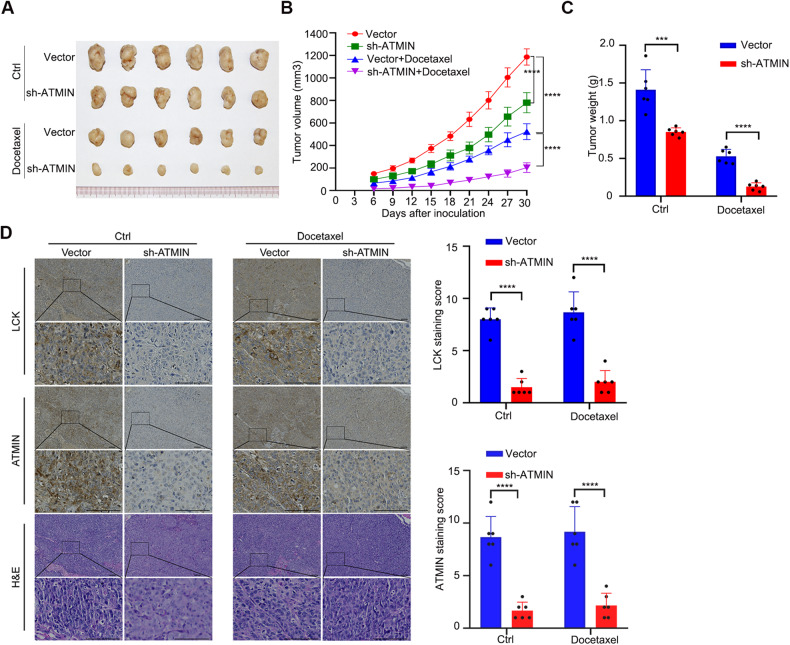
Knockdown of ATMIN suppresses tumor growth and enhances docetaxel-sensitivity of NPC in vivo. SUNE1 cells with or without ATMIN silencing were transplanted into the right axillary region of nude mice. Once the tumor nodes became palpable (~100 mm^3^), the mice were randomly divided into four groups (*n* = 6 per group) and intraperitoneally injected with saline or docetaxel every three days. **A** Representative images of the xenograft tumors. **B** Tumor volume growth curves of the xenograft tumors. **C** Weight of xenograft tumors in the saline group (left) and docetaxel group (right). **D** Representative images of H&E and IHC staining and staining scores of ATMIN and LCK in subcutaneous tumors from different groups. Data are presented as mean ± SD, *P* values were calculated using two-way ANOVA (**B**) or Student’s *t* test (**C**, **D**). ***P < 0.001, ****P < 0.0001.

To further validate the function of ATMIN in vivo, we injected SUNE1 cells with or without stable knockdown of ATMIN into nude mice and administrated with docetaxel to construct a subcutaneous tumor xenograft model. Compared with the control group, the ATMIN-knockdown group exhibited reduced xenograft growth in terms of the tumor volume, growth rate and weight, especially after docetaxel administration, indicating that the tumors in the ATMIN-knockdown group were more sensitive to docetaxel (Fig. 6A–C). To further determine whether ATMIN regulates the expression of LCK in vivo, IHC staining of ATMIN and LCK was performed in these tumors. Consistently, the expression of LCK was significantly decreased in the ATMIN-knockdown group with or without docetaxel treatment (Fig. 6D). Collectively, these results confirm that knockdown of ATMIN suppresses NPC tumor growth and enhances docetaxel sensitivity of NPC in vivo.

## Discussion

In the present study, we identified ATMIN as a chemoresistance gene in response to TPF chemotherapy in NPC patients and confirmed that ATMIN promotes docetaxel-resistance and tumor growth both in vitro and in vivo. Moreover, we identified USP10 as a potent DUB for ATMIN protein stabilization. We further confirmed that ATMIN is associated with the cell death signaling and revealed that ATMIN transcriptionally activates LCK to facilitate the proliferation and chemoresistance of NPC cells.

ATMIN regulates biological functions in a variety of cancers. In ovarian cancer, ATMIN promotes cisplatin resistance by regulating the DYNLL1-MRN signaling axis [39]; in tongue cancer, ATMIN promotes tumor metastasis by activating KRAS pathway [40], while its role in NPC remains to be investigated. Here, we have for the first time found that ATMIN promotes docetaxel resistance and tumor proliferation in NPC, which may provide a novel therapeutic target for the management of advanced NPC.

The ubiquitin-proteasome system is one of the important ways for the selective protein degradation in eukaryotes, which is widely involved in numerous physiological processes including apoptosis, antigen presentation, and intracellular signaling [41]. In this system, substrate proteins are specifically recognized by the ubiquitinases which bind ubiquitin chains to the target substrate proteins, and subsequently the proteases catalyze degradation of the ubiquitinated substrate proteins. Conversely, deubiquitinases (DUBs) can remove ubiquitin chains bound to the substrate proteins, thereby inhibiting their degradation [42]. At present, about 103 DUBs have been identified in the human genome, which can be divided into six families: ubiquitin-specific proteases, ubiquitin c-terminal hydrolases, ovarian oncoproteins, MJD, MINDY, and JAMM [43]. Among them, the most widely studied is the ubiquitin-specific protease family, which was found to play a crucial regulatory role in the development, treatment resistance, occurrence, and metastasis of tumors. USP10 belongs to the ubiquitin-specific protease family. Previous studies have reported that USP10 promotes cisplatin resistance by deubiquitinating HDAC6 in non-small cell lung cancer [44], and promotes tumor growth and proliferation by deubiquitinating TAZ/YAP in liver cancer [45]. In this study, we found that USP10 can deubiquitinate and thus stabilize ATMIN protein and may be one of the possible mechanisms for the up-regulation of ATMIN expression in NPC.

ATMIN functions as a transcription factor mainly in two ways: one is that it combines with ATM, and then phosphorylates downstreams to participate in oxidative stress and DNA damage repair [18, 19]; the other one is that it combines with the promoter regions of target genes to promote their transcriptions and regulate the growth and development of cells and organs [20]. Here, we screened and identified ten candidate genes regulated by ATMIN. Among them, several genes have been reported to play critical roles in tumor progression. For instance, CYTH4 has been reported to be up-regulated and associated with worse survival in ovarian cancer and glioma [28, 29]. Dey et al. reported that LCK facilitates DNA damage repair and hampers the efficacy of PARPi in ovarian cancer [30]. Huang et al. reported that LCK regulates TRPM8 assembly to promote pancreatic cancer malignancy [31]. It has been found that FKBP1A is up-regulated in prostate cancer and head and neck squamous cell carcinoma, and enhances paclitaxel-resistance and associates with lymph node metastasis [32, 33]. Moreover, Zhang et al. reported that DUSP2 promotes apoptosis under hypoxic microenvironment in pancreatic cancer [34]. Ding at el. revealed that downregulation of DUSP2 by DNTTIP1 promotes metastasis in NPC [35]. Since ATMIN could transcriptionally regulate these genes in NPC, we speculate that ATMIN may also exert oncogenic functions in NPC through these downstream genes. However, only the expression correlation between LCK and ATMIN in NPC patients is consistent with the RNA-seq and the qRT-PCR results, leading us to choose LCK for further investigation. LCK belongs to the Src family tyrosine kinases, participating in the regulation of cell cycle, apoptosis and differentiation by maintaining the AKT and MAPK pathways [46]. Previous studies reported that LCK is an oncogene and the use of LCK inhibitors can inhibit glioma growth, metastasis and the expression of cell stemness-related genes [37], and enhance cisplatin sensitivity in ovarian cancer [38]. Our results found that ATMIN promotes docetaxel resistance and growth and proliferation through transcriptional activation of LCK in NPC, and overexpression of LCK could reverse the effects of ATMIN-knockdown-mediated cell proliferation suppression and docetaxel sensitization in NPC cells, showing that LCK is a functional downstream target gene of ATMIN in NPC.

Despite our findings about ATMIN in chemoresistance in NPC, there are still a lot of work that need to be done in the future. For example, IC regimens for advanced NPC includes TPF, TP (docetaxel + cisplatin) and GP (gemcitabine + cisplatin) regimens in clinical practice [47]. This study has revealed the effect of ATMIN on the sensitivity of TPF-based IC, whether ATMIN is also involved in regulating the sensitivity of gemcitabine or other drugs in NPC needs to be further verified.

In conclusions, our findings identified ATMIN as a chemoresistance gene in response to TPF chemotherapy in NPC patients. ATMIN promotes docetaxel-resistance and tumor growth in NPC cells. Mechanistically, we identified USP10 as a potent DUB for ATMIN protein stabilization. We further confirmed that ATMIN is associated with the cell death signaling and it transcriptionally activates LCK to facilitate the proliferation and chemoresistance of NPC. Our findings broaden the insights into the chemoresistance mechanism of NPC and the USP10-ATMIN-LCK axis provides potential therapeutic targets for the management of advanced NPC.

## Methods

### Cell culture and treatment

The immortalized normal human nasopharyngeal epithelial cell line NP69 and human NPC cell lines (HONE1, SUNE1, CNE1, HNE1, HK1 and C666-1) were generously given by Professor Mu-Sheng Zeng from Sun Yat-sen University Cancer Center, Guangzhou, China. Keratinocyte serum-free medium (Invitrogen) containing 10% bovine pituitary extract (BDBiosciences) was used for culturing NP69, while RPMI-1640 medium (Gibco) containing 10% fetal bovine serum (FBS, Gibco) was used for culturing NPC cell lines. DMEM medium (Gibco) containing 10% FBS was used for culturing HEK293T cells, which were acquired from the American Type Tissue Culture Collection (ATCC).

To inhibit the ubiquitin-proteasome-dependent protein degradation, cells were treated with 10 μM MG132 for 6 h. To inhibit the lysosome-dependent protein degradation, cells were treated with 50 μM chloroquine (CQ, Sigma-Aldrich) for 6 h. For the inhibition of protein synthesis, cells were treated with 100 μg/mL cycloheximide (CHX, Sigma) for 0, 3, 6, 9, or 12 h.

### Plasmid construction and transfection

The following plasmids were constructed by cloning the coding sequences of USP10, ATMIN and LCK that are tagged with MYC, HA, or FLAG into the empty vector: pSin-EF2-puro-USP10-MYC, pSin-EF2-puro-USP10-HA, pSin-EF2-puro-ATMIN-FLAG, pSin-EF2-puro-ATMIN-HA, and pcDNA3.4-LCK-EYFP-3X FLAG. Meanwhile, the pCMV-kana-Ub (WT)-HA plasmid was acquired from Vigene Bioscience (China), and the USP10 siRNA (si-USP10) and ATMIN siRNA (si-ATMIN) were purchased from RiboBio (China). The targeted sequences of the used siRNA were as follows: si-USP10#1: 5′-GCTTCTCTCACCAAGTAAT-3′; si-USP10#2: 5′-GGTGGCCTATGTGGAAACTAAGTAT-3′; si-ATMIN#1: 5′-GGAACACGATGGAGTCTCA-3′; si-ATMIN#2: 5′-CTACTGCTGTCCAATTGAA-3′. The shRNA sequence targeting ATMIN was consistent with the si-ATMIN targeting sequence and was inserted into the pLKO.1-RFP vector to obtain the PLKO.1-shATMIN plasmid. Transfections were conducted using NEOFECT^TM^ DNA transfection reagent or Lipofectamine 3000 (Invitrogen) under the manufacturer’s instruction. The efficiency of transfection was tested by qRT-PCR or western blot, or both after 24–48 h of transfection.

### qRT-PCR

TRIzol reagent (Invitrogen) was used for extracting total RNA according to the manufacturer’s protocol. GoScript™ Reverse Transcription System kit (Promega) was used for synthesizing complementary DNA. qPCR was performed with ChamQ SYBR qPCR Master Mix (Vazyme) on a CFX96 Touch sequence detection system (Bio-Rad). The 2-ΔΔCT method was adopted for calculating gene expression, with *GAPDH* expression set as the internal control. Supplementary Table S1 summarizes the primer sequences used for qPCR.

### Western blot analysis

1 × RIPA lysis buffer (Millipore) containing both protease and phosphatase inhibitor (Roche) was used for cell lysis. The lysates were subjected to sonication to acquire total protein, which was subsequently separated by SDS–PAGE and transferred to PVDF membranes (Millipore). After blocking with 5% nonfat powdered milk for 1 h, the membranes were processed first with primary antibodies at 4 °C overnight and then with secondary antibodies at room temperature for 1 h. Supplementary Table S2 lists the antibodies used.

### Cell viability assay and chemotherapy drug treatment

800 HONE1 cells or 1000 SUNE1 cells per well were seeded into the 96-well plates. On the indicated days from day 1 to day 5, 10 μl Cell Counting Kit-8 (CCK-8) reagent (TargetMol) per well was added to the 96-well plates. The absorbance at 450 nm for each well was measured by a spectrophotometer after incubation at 37 °C for 2 h.

In addition, 2000 HONE1 cells or 3000 SUNE1 cells per well were plated into the 96-well plates, and were treated with docetaxel, cisplatin and 5-FU with the indicated concentration, respectively. After 48 hours, the 96-well plates were subjected to the cell viability assay as mentioned above.

### Clonogenic assay

800 HONE1 cells or 1000 SUNE1 cells per well were seeded into the six-well plates, and were cultured until single-cell colonies formed. Cells were processed with methanol for 1 h and then stained with crystal violet for 10 min.

### Co-IP assay

Cells were lysed on ice with IP lysis buffer containing both protease and phosphatase inhibitor. The obtained lysates were incubated with the indicated antibodies at 4 °C overnight. The immune complexes were obtained using Pierce^TM^ Protein A/G Magnetic Beads (Thermo Scientific) and were then washed with IP wash buffer for five times. The obtained eluates were subjected to SDS–PAGE and then Fast Silver Stain Kit (Beyotime) for staining. The proteins of interest acquired by Co-IP were subjected to western blot or mass spectrometry analysis, which was performed by FITGENE (Guangzhou, China). Supplementary Table S2 lists the antibodies used and the mass spectrometry analysis data is provided in Supplementary Table S3.

### Immunofluorescence assay

Cells were sequentially processed with 0.4% paraformaldehyde for fixation, 0.5% Triton X-100 for permeabilization, and QuickBlock™ Blocking Buffer for Immunol Staining (Beyotime) for blocking nonspecific binding. Then, the cells were incubated with primary antibodies at 4 °C overnight and stained with secondary antibodies and 4’,6-diamidino-2-phenylindole (DAPI, Sigma) at room temperature with the indicated time. Fluorescence images were obtained with an LSM880 confocal scanning microscope. Supplementary Table S2 summarizes the antibodies used.

### RNA sequencing

After HONE1 cells were transfected with si-ATMIN or control for 48 h, total RNA was extracted and used for sequencing library construction. RNA-seq was conducted on the NovaSeq 6000 (Illumina) platform by Nobelio Biotechnology (Guangzhou, China). Gene Oncology (GO) and Kyoto Encyclopedia of Genes and Genomes (KEGG) pathway analysis were performed based on the differentially expressed genes upon ATMIN knockdown with the DAVID software (https://david.ncifcrf.gov/).

### Chromatin immunoprecipitation (ChIP)

Pierce Magnetic ChIP Kit (Thermo Fisher Scientific) was used for ChIP assay according to the protocols. In brief, cells were cross-linked with 1% formaldehyde, quenched with 125 mM glycine, and then lysed and sonicated to obtain chromatin fragments. The obtained chromatin fragments were immunoprecipitated with the indicated antibody. DNA was isolated and ChIP-seq was performed on the NovaSeq 6000 (Illumina) platform by Nobelio Biotechnology (Guangzhou, China). The input DNA and ChIP with anti-IgG antibody were used for normalization and Supplementary Table S1 lists the primers used for ChIP-PCR.

### Luciferase reporter assay

The sequence of *LCK* promoter was inserted into the pGL3-Basic vector. Cells were plated into six-well plates and transfected with the constructed pGL3-Basic-LCK plasmid, together with pSin-EF2-puro-ATMIN-FLAG plasmid or its empty vector. The firefly and the renilla luciferase activities were detected with the Dual-Luciferase Reporter Assay System (Promega) for each well under the manufacturer’s instruction, with renilla luciferase activity used for internal control.

### Murine xenograft growth of NPC

Female BALB/c nude mice of 6–8 weeks old were obtained from Charles River Laboratories (Beijing, China) and were administered a subcutaneous injection of 1 × 10^6^ SUNE1 cells with or without stably ATMIN-knockdown. The mice were monitored every three days after 6 days of tumor injection, tumor volume and body weight were measured and recorded. Tumor volumes were calculated with the following formula: 0.5 × length × width^2^. When the volume of xenograft tumors reached approximately 100 mm^3^, the mice were intraperitoneally injected with docetaxel (10 mg/kg) or saline every three days. After 30 days of tumor injection, tumor samples were harvested and paraffin embedded.

### Immunohistochemistry (IHC)

Tumor paraffin-embedded sections were successively processed with dimethylbenzene and ethanol for deparaffinization, 3% H_2_O_2_ for inactivation of endogenous peroxidase activity, citrate buffer solution for antigen retrieval, and QuickBlock™ Blocking Buffer for Immunol Staining (Beyotime) for blocking nonspecific binding. The processed sections were incubated first with primary antibody at 4 °C overnight and then secondary antibody at room temperature for 30 min. DAB Detection Kit (ZSGB-BIO) was used for staining. Images of the stained tumor sections were obtained with the DAKO REAL EnVision Inspection System.

### Statistical analysis

Student’s *t* test, Wilcoxon rank sum test and one-way or two-way analysis of variance (ANOVA) were used to compare continuous variables between groups. Survival data was plotted with Kaplan–Meier curves and the survival differences were determined by log-rank test. GraphPad Prism (version 8) was used for statistical analysis. When *P*-value was < 0.05, results were considered as statistically significant.

### Supplementary information

Supplementary material

Original western blots

Reproducibility Checklist

## Data Availability

The RNA-seq and ChIP-seq profiles are accessible at the GEO repository with the accession number GSE236505. The key raw data of this study were uploaded to the Research Data Deposit public platform (http://www.researchdata.org.cn, accession number: RDDB2023651620) and are available upon reasonable request to the corresponding authors.
