# Supplementary material for: Transcription factor ATMIN facilitates chemoresistance in nasopharyngeal carcinoma
Source: Cell Death Dis. 2024 Feb 6;15(2):112. doi: 10.1038/s41419-024-06496-x (PMC10847093; doi:10.1038/s41419-024-06496-x)

**Supplementary Information**

**Transcription factor ATMIN facilitates chemoresistance in nasopharyngeal carcinoma**

Xue-Liang Fang^1,#^, Qing-Jie Li^1,#^, Jia-Yi Lin^1,#^, Cheng-Long Huang^1^, Sheng-Yan Huang^1^, Xi-Rong Tan^1^, Shi-Wei He^1^, Xun-Hua Zhu^1^, Jun-Yan Li^1^, Sha Gong^1^, Han Qiao^1^, Ying-Qin Li^1^; Na Liu^1^; Jun Ma^1^, Yin Zhao^1,*^, Ling-Long Tang^1,*^

**Supplementary Table S1. Primer sequences used in the study.**

|  | **Forward Primer (5′→3′)** | **Reverse Primer (5′→3′)** |
| --- | --- | --- |
| **Used for**  **qRT–PCR** |  |  |
| *ATMIN* | CGCGCTCAACATGCACCTA | GCAGTAGAATTTCGGTCCAGTT |
| *FKBP1A* | CTCCAGATTATGCCTATGGTGC | AGCTCCACATCGAAGACGAGA |
| *CYP11A1* | GCAGTGTCTCGGGACTTCG | GGCAAAGCGGAACAGGTCA |
| *KALRN* | TGTTGCTTCCCGTGTAGAGAT | GTCAAAGCTCTTCCGACCATC |
| *EIF4EBP2* | TAGCCCTGGCACCTTAATTGA | ATCCCCAACTGCATGTTTCCT |
| *DUSP2* | GGGCTCCTGTCTACGACCA | GCAGGTCTGACGAGTGACTG |
| *SSUH2* | CCAGCTTGTCATCATGTGGAAG | CAGCAATACCGCTCAGGATAGTC |
| *PSTPIP1* | TATGAGGCCGTCATGGACC | GCACTTCTGCTCGTATGTCTT |
| *CYTH4* | GAGACGGAAGAGTTACAGAGGA | AGTCGATTTGGGCAAACACAT |
| *NAV3* | AGCCTGTGCATACTGCTCTTC | TGATTTTAACGCAAGCTGACAAG |
| *LCK* | TGCCATTATCCCATAGTCCCA | GAGCCTTCGTAGGTAACCAGT |
| *GAPDH* | GGAGCGAGATCCCTCCAAAAT | GGCTGTTGTCATACTTCTCATGG |
| **Used for**  **ChIP-PCR** |  |  |
| *LCK* | ATGTCCCCGGACGCCTTC | AGTATCCCCTGGTAGCCGAC |

**Supplementary Table S2. Antibodies used in the study.**

| **Antibody** | **Company** | **Catalogue No.** | **Application** | **Dilution** |
| --- | --- | --- | --- | --- |
| Used for Western blotting (WB) and immunoprecipitation (IP) | | | | |
| anti-ATMIN | Bethyl | A303-399A | WB, IP | 1:1000 for WB, 3ug for IP |
| anti-USP10 | Proteintech | 19374-1-AP | WB, IP | 1:1000 for WB, 3ug for IP |
| anti-HA | Sigma–Aldrich | H6908 | WB, IP | 1:1000 for WB, 3ug for IP |
| anti-LCK | Abcam | Ab227976 | WB | 1:1000 |
| anti-FLAG | Sigma–Aldrich | F3165 | IP | 3ug for IP |
| anti-MYC | Proteintech | 16286-1-AP | IP | 3ug for IP |
| anti-GAPDH | Abcam | ab8245 | WB | 1:5000 |
| anti-α-tubulin | Abcam | 66031-1-Ig | WB | 1:5000 |
| anti-rabbit IgG | CST | 7074 | WB | 1:10000 |
| anti-mouse IgG | CST | 7076 | WB | 1:10000 |
| Used for immunofluorescence (IF) and immunohistochemical (IHC) staining | | | | |
| anti-ATMIN | Abnova | H00023300-B01P | IF | 1:500 |
| anti-USP10 | Proteintech | 19374-1-AP | IF | 1:500 |
| Alexa Fluor 488 | Thermo Fisher Scientific | A11001 | IF | 1:2000 |
| Alexa Fluor 594 | Thermo Fisher Scientific | A11012 | IF | 1:2000 |
| anti-ATMIN | CUSABIO | CSB-PA002304ESRHU | IHC | 1:200 |
| anti-LCK | Abcam | Ab227976 | IHC | 1:200 |

**Supplementary Table S3. Mass spectrometry analysis results for the anti-FLAG (FLAG-tagged ATMIN) immunoprecipitation complex.**

| **Accession** | **Protein description** | **Score** | **Matches** |
| --- | --- | --- | --- |
| ATMIN_HUMAN | ATM interactor | 1354 | 43 |
| ATD3A_HUMAN | ATPase family AAA domain-containing protein 3A | 549 | 21 |
| ATD3B_HUMAN | ATPase family AAA domain-containing protein 3B | 400 | 16 |
| TOP2B_HUMAN | DNA topoisomerase 2-beta | 274 | 8 |
| EFTU_HUMAN | Elongation factor Tu, mitochondrial | 259 | 7 |
| AT1A1_HUMAN | Sodium/potassium-transporting ATPase subunit alpha-1 | 257 | 7 |
| TIF1B_HUMAN | Transcription intermediary factor 1-beta | 231 | 6 |
| HNRH1_HUMAN | Heterogeneous nuclear ribonucleoprotein H | 227 | 6 |
| DNLI3_HUMAN | DNA ligase 3 | 224 | 11 |
| NOP58_HUMAN | Nucleolar protein 58 | 215 | 4 |
| ELYS_HUMAN | Protein ELYS | 213 | 5 |
| BCLF1_HUMAN | Bcl-2-associated transcription factor 1 | 210 | 4 |
| YBOX3_HUMAN | Y-box-binding protein 3 | 210 | 3 |
| RL4_HUMAN | 60S ribosomal protein L4 | 210 | 11 |
| CBP_HUMAN | CREB-binding protein | 206 | 6 |
| RPN1_HUMAN | Dolichyl-diphosphooligosaccharide-protein  glycosyltransferase subunit 1 | 193 | 6 |
| MOV10_HUMAN | Helicase MOV-10 | 182 | 3 |
| TR150_HUMAN | Thyroid hormone receptor-associated protein 3 | 178 | 4 |
| RBM39_HUMAN | RNA-binding protein 39 | 178 | 5 |
| RACK1_HUMAN | Receptor of activated protein C kinase 1 | 170 | 5 |
| ASPH_HUMAN | Aspartyl/asparaginyl beta-hydroxylase | 163 | 4 |
| IF2B_HUMAN | Eukaryotic translation initiation factor 2 subunit 2 | 159 | 4 |
| TKT_HUMAN | Transketolase | 158 | 2 |
| RFC1_HUMAN | Replication factor C subunit 1 | 157 | 3 |
| RL10_HUMAN | 60S ribosomal protein L10 | 153 | 8 |
| NU107_HUMAN | Nuclear pore complex protein Nup107 | 150 | 3 |
| DHX15_HUMAN | Pre-mRNA-splicing factor ATP-dependent RNA helicase DHX15 | 150 | 4 |
| IMA1_HUMAN | Importin subunit alpha-1 | 149 | 3 |
| SRPK1_HUMAN | SRSF protein kinase 1 | 148 | 3 |
| FAS_HUMAN | Fatty acid synthase | 143 | 4 |
| CD109_HUMAN | CD109 antigen | 142 | 2 |
| NU153_HUMAN | Nuclear pore complex protein Nup153 | 142 | 7 |
| SMCA5_HUMAN | SWI/SNF-related matrix-associated actin-dependent regulator of chromatin subfamily A member 5 | 141 | 5 |
| SRSF2_HUMAN | Serine/arginine-rich splicing factor 2 | 136 | 2 |
| RS16_HUMAN | 40S ribosomal protein S16 | 135 | 5 |
| BAG3_HUMAN | BAG family molecular chaperone regulator 3 | 134 | 4 |
| RUVB1_HUMAN | RuvB-like 1 | 134 | 4 |
| ILF2_HUMAN | Interleukin enhancer-binding factor 2 | 134 | 5 |
| COPA_HUMAN | Coatomer subunit alpha | 131 | 7 |
| SSRP1_HUMAN | FACT complex subunit SSRP1 | 129 | 3 |
| PO210_HUMAN | Nuclear pore membrane glycoprotein 210 | 129 | 5 |
| LC7L2_HUMAN | Putative RNA-binding protein Luc7-like 2 | 128 | 3 |
| SUN1_HUMAN | SUN domain-containing protein 1 | 127 | 3 |
| MIC60_HUMAN | MIC complex subunit MIC60 | 126 | 6 |
| EZRI_HUMAN | Ezrin | 121 | 3 |
| DHX30_HUMAN | ATP-dependent RNA helicase DHX30 | 121 | 5 |
| RCN1_HUMAN | Reticulocalbin-1 | 120 | 3 |
| ITB4_HUMAN | Integrin beta-4 | 120 | 2 |
| TRIP6_HUMAN | Thyroid receptor-interacting protein 6 | 119 | 2 |
| GTR1_HUMAN | Solute carrier family 2, facilitated glucose transporter member 1 | 119 | 4 |
| KANK2_HUMAN | KN motif and ankyrin repeat domain-containing protein 2 | 117 | 4 |
| GCN1_HUMAN | eIF-2-alpha kinase activator GCN1 | 116 | 5 |
| ACINU_HUMAN | Apoptotic chromatin condensation inducer in the nucleus | 115 | 2 |
| FINC_HUMAN | Fibronectin | 114 | 4 |
| HNRPF_HUMAN | Heterogeneous nuclear ribonucleoprotein F | 114 | 4 |
| PDS5B_HUMAN | Sister chromatid cohesion protein PDS5 homolog B | 113 | 3 |
| RRP44_HUMAN | Exosome complex exonuclease RRP44 | 112 | 3 |
| CPSM_HUMAN | Carbamoyl-phosphate synthase [ammonia], mitochondrial | 110 | 3 |
| FACR1_HUMAN | Fatty acyl-CoA reductase 1 | 110 | 3 |
| MCM3_HUMAN | DNA replication licensing factor MCM3 | 109 | 4 |
| RUVB2_HUMAN | RuvB-like 2 | 108 | 6 |
| SFXN1_HUMAN | Sideroflexin-1 | 107 | 2 |
| EMD_HUMAN | Emerin | 107 | 3 |
| P3C2A_HUMAN | Phphatidylinitol 4-phphate 3-kinase C2 domain-containing subunit alpha | 106 | 3 |
| SYMC_HUMAN | Methionine--tRNA ligase, cytoplasmic | 106 | 2 |
| NUP93_HUMAN | Nuclear pore complex protein Nup93 | 104 | 5 |
| SRPRB_HUMAN | Signal recognition particle receptor subunit beta | 104 | 2 |
| UBP10_HUMAN | Ubiquitin carboxyl-terminal hydrolase 10 | 104 | 4 |
| KI67_HUMAN | Proliferation marker protein Ki-67 | 104 | 4 |
| COPB2_HUMAN | Coatomer subunit beta | 103 | 3 |
| FBN2_HUMAN | Fibrillin-2 | 102 | 2 |
| TIM23_HUMAN | Mitochondrial import inner membrane translocase subunit Tim23 | 102 | 2 |
| ERLN2_HUMAN | Erlin-2 | 101 | 3 |
| E41L2_HUMAN | Band 4.1-like protein 2 | 101 | 2 |
| EP300_HUMAN | Histone acetyltransferase p300 | 99 | 5 |
| RSSA_HUMAN | 40S ribosomal protein SA | 98 | 1 |
| TXTP_HUMAN | Tricarboxylate transport protein, mitochondrial | 97 | 4 |
| PP1A_HUMAN | Serine/threonine-protein ph phatase PP1-alpha catalytic subunit | 97 | 5 |
| LYAR_HUMAN | Cell growth-regulating nucleolar protein | 97 | 2 |
| PDIA1_HUMAN | Protein disulfide-isomerase | 96 | 1 |
| CPT1A_HUMAN | Carnitine O-palmitoyltransferase 1, liver isoform | 96 | 3 |
| PRP8_HUMAN | Pre-mRNA-processing-splicing factor 8 | 96 | 4 |
| RCN2_HUMAN | Reticulocalbin-2 | 95 | 2 |
| PGAM5_HUMAN | Serine/threonine-protein phphatase PGAM5, mitochondrial | 95 | 3 |
| COR1B_HUMAN | Coronin-1B | 94 | 3 |
| TCPG_HUMAN | T-complex protein 1 subunit gamma | 94 | 2 |
| SND1_HUMAN | Staphylococcal nuclease domain-containing protein 1 | 93 | 2 |
| M2OM_HUMAN | Mitochondrial 2-oxoglutarate/malate carrier protein | 92 | 3 |
| RAD50_HUMAN | DNA repair protein RAD50 | 92 | 3 |
| SF3B3_HUMAN | Splicing factor 3B subunit 3 | 91 | 3 |
| SPTC1_HUMAN | Serine palmitoyltransferase 1 | 90 | 1 |
| SON_HUMAN | Protein SON | 89 | 2 |
| 1433Z_HUMAN | 14-3-3 protein zeta/delta | 89 | 1 |
| RBM4_HUMAN | RNA-binding protein 4 | 88 | 1 |
| ZFR_HUMAN | Zinc finger RNA-binding protein | 88 | 1 |
| IF2B3_HUMAN | Insulin-like growth factor 2 mRNA-binding protein 3 | 87 | 1 |
| NAT10_HUMAN | RNA cytidine acetyltransferase | 87 | 3 |
| RCC1_HUMAN | Regulator of chromome condensation | 87 | 1 |
| NXF1_HUMAN | Nuclear RNA export factor 1 | 86 | 1 |
| EIF3F_HUMAN | Eukaryotic translation initiation factor 3 subunit F | 86 | 1 |
| RS5_HUMAN | 40S ribosomal protein S5 | 86 | 3 |
| SLAI2_HUMAN | SLAIN motif-containing protein 2 | 85 | 1 |
| ATPMD_HUMAN | ATP synthase membrane subunit DAPIT, mitochondrial | 85 | 2 |
| AP2B1_HUMAN | AP-2 complex subunit beta | 85 | 5 |
| ABLM1_HUMAN | Actin-binding LIM protein 1 | 85 | 2 |
| CPSF1_HUMAN | Cleavage and polyadenylation specificity factor subunit 1 | 85 | 2 |
| WDR11_HUMAN | WD repeat-containing protein 11 | 84 | 1 |
| LC7L3_HUMAN | Luc7-like protein 3 | 84 | 2 |
| PYR1_HUMAN | CAD protein | 84 | 2 |
| STRAP_HUMAN | Serine-threonine kinase receptor-associated protein | 84 | 2 |
| TSYL2_HUMAN | Testis-specific Y-encoded-like protein 2 | 84 | 1 |
| GGYF2_HUMAN | GRB10-interacting GYF protein 2 | 84 | 3 |
| BAG2_HUMAN | BAG family molecular chaperone regulator 2 | 83 | 2 |
| P4HA1_HUMAN | Prolyl 4-hydroxylase subunit alpha-1 | 82 | 1 |
| HDAC1_HUMAN | Histone deacetylase 1 | 81 | 3 |
| DRG1_HUMAN | Developmentally-regulated GTP-binding protein 1 | 81 | 2 |
| YMEL1_HUMAN | ATP-dependent zinc metalloprotease YME1L1 | 81 | 2 |
| RS13_HUMAN | 40S ribosomal protein S13 | 81 | 2 |
| DNJC9_HUMAN | DnaJ homolog subfamily C member 9 | 81 | 3 |
| HSPB1_HUMAN | Heat shock protein beta-1 | 80 | 3 |
| AKP13_HUMAN | A-kinase anchor protein 13 | 80 | 1 |
| RS2_HUMAN | 40S ribosomal protein S2 | 79 | 6 |
| SPB1_HUMAN | pre-rRNA 2-O-ribose RNA methyltransferase FTSJ3 | 79 | 2 |
| H2A3_HUMAN | Histone H2A type 3 | 79 | 6 |
| PFKAP_HUMAN | ATP-dependent 6-phosphofructokinase, platelet type | 79 | 1 |
| SMC1A_HUMAN | Structural maintenance of chromomes protein 1A | 79 | 5 |
| RLA0L_HUMAN | 60S acidic ribosomal protein P0-like | 78 | 3 |
| SMCE1_HUMAN | SWI/SNF-related matrix-associated actin-dependent regulator of chromatin subfamily E member 1 | 78 | 1 |
| ZC11A_HUMAN | Zinc finger CCCH domain-containing protein 11A | 78 | 2 |
| DHB11_HUMAN | Estradiol 17-beta-dehydrogenase 11 | 78 | 1 |
| QCR2_HUMAN | Cytochrome b-c1 complex subunit 2, mitochondrial | 78 | 2 |
| ANKZ1_HUMAN | Ankyrin repeat and zinc finger domain-containing  protein 1 | 78 | 1 |
| EF1D_HUMAN | Elongation factor 1-delta | 78 | 2 |
| STAU1_HUMAN | Double-stranded RNA-binding protein Staufen homolog 1 | 78 | 2 |
| H1X_HUMAN | Histone H1.10 | 77 | 2 |
| GANAB_HUMAN | Neutral alpha-glucosidase AB | 77 | 1 |
| SF3B1_HUMAN | Splicing factor 3B subunit 1 | 77 | 2 |
| ASCC3_HUMAN | Activating signal cointegrator 1 complex subunit 3 | 77 | 3 |
| SQOR_HUMAN | Sulfide:quinone oxidoreductase, mitochondrial | 76 | 2 |
| PSA5_HUMAN | Proteasome subunit alpha type-5 | 75 | 2 |
| TCPQ_HUMAN | T-complex protein 1 subunit theta | 74 | 3 |
| EXOS7_HUMAN | Exome complex component RRP42 | 74 | 1 |
| CXG1_HUMAN | Gap junction gamma-1 protein | 74 | 1 |
| CTNA1_HUMAN | Catenin alpha-1 | 73 | 1 |
| OASL_HUMAN | 2'-5'-oligoadenylate synthase-like protein | 73 | 1 |
| RS9_HUMAN | 40S ribosomal protein S9 | 73 | 5 |
| GCR_HUMAN | Glucocorticoid receptor | 73 | 2 |
| LAMC2_HUMAN | Laminin subunit gamma-2 | 73 | 1 |
| NU205_HUMAN | Nuclear pore complex protein Nup205 | 72 | 2 |
| RL23_HUMAN | 60S ribosomal protein L23 | 71 | 1 |
| RS24_HUMAN | 40S ribosomal protein S24 | 70 | 1 |
| CCD86_HUMAN | Coiled-coil domain-containing protein 86 | 70 | 1 |
| TES_HUMAN | Testin | 69 | 2 |
| SPA5L_HUMAN | Spermatogenesis-associated protein 5-like protein 1 | 69 | 1 |
| U5S1_HUMAN | 116 kDa U5 small nuclear ribonucleoprotein component | 69 | 3 |
| C1TC_HUMAN | C-1-tetrahydrofolate synthase, cytoplasmic | 68 | 1 |
| SPAS2_HUMAN | Spermatogenesis-associated serine-rich protein 2 | 68 | 3 |
| LAS1L_HUMAN | ribosomal biogenesis protein LAS1L | 68 | 1 |
| TOM22_HUMAN | Mitochondrial import receptor subunit TOM22 homolog | 68 | 1 |
| NTPCR_HUMAN | Cancer-related nucle ide-triph phatase | 67 | 1 |
| SRSF3_HUMAN | Serine/arginine-rich splicing factor 3 | 67 | 3 |
| FBRL_HUMAN | rRNA 2-O-methyltransferase fibrillarin | 67 | 2 |
| DDX27_HUMAN | Probable ATP-dependent RNA helicase DDX27 | 66 | 4 |
| FA83H_HUMAN | Protein FAM83H | 66 | 1 |
| ESYT2_HUMAN | Extended synaptotagmin-2 | 66 | 2 |
| RS26_HUMAN | 40S ribosomal protein S26 | 66 | 4 |
| RAB10_HUMAN | Ras-related protein Rab-10 | 66 | 2 |
| GPAT3_HUMAN | Glycerol-3-phosphate acyltransferase 3 | 65 | 1 |
| STOM_HUMAN | Stomatin | 64 | 2 |
| S61A1_HUMAN | Protein transport protein Sec61 subunit alpha isoform 1 | 64 | 3 |
| RBM4B_HUMAN | RNA-binding protein 4B | 64 | 1 |
| MECP2_HUMAN | Methyl-CpG-binding protein 2 | 63 | 1 |
| SYEP_HUMAN | Bifunctional glutamate/proline-tRNA ligase | 63 | 2 |
| PIAS1_HUMAN | E3 SUMO-protein ligase PIAS1 | 63 | 1 |
| TMM33_HUMAN | Transmembrane protein 33 | 63 | 3 |
| FKBP8_HUMAN | Peptidyl-prolyl cis-trans isomerase FKBP8 | 63 | 1 |
| PSMD3_HUMAN | 26S proteasome non-ATPase regulatory subunit 3 | 63 | 2 |
| CG050_HUMAN | Uncharacterized protein C7orf50 | 62 | 1 |
| ARHG2_HUMAN | Rho guanine nucleotide exchange factor 2 | 62 | 2 |
| ACOD_HUMAN | Acyl-CoA desaturase | 62 | 1 |
| RRP12_HUMAN | RRP12-like protein | 62 | 1 |
| NMD3_HUMAN | 60S ribosomal export protein NMD3 | 62 | 1 |
| PLCE_HUMAN | 1-acyl-sn-glycerol-3-phosphate acyltransferase epsilon | 62 | 1 |
| H2AZ_HUMAN | Histone H2A.Z | 62 | 5 |
| RBM10_HUMAN | RNA-binding protein 10 | 62 | 2 |
| TDIF2_HUMAN | Deoxynucleotidyltransferase terminal-interacting protein 2 | 61 | 1 |
| ANR17_HUMAN | Ankyrin repeat domain-containing protein 17 | 61 | 2 |
| SSRG_HUMAN | Translocon-associated protein subunit gamma | 60 | 1 |
| PHIP_HUMAN | PH-interacting protein | 60 | 3 |
| CHTOP_HUMAN | Chromatin target of PRMT1 protein | 60 | 1 |
| FAF2_HUMAN | FAS-associated factor 2 | 60 | 1 |

**Supplementary Fig. S1.** ATMIN mRNA expression in NPC patients with response (n=48) and non-response (n=12) to TPF chemotherapy in the Guilin dataset. Data are presented as mean ± SD, *P* value was calculated using Student’s t test. ***P*<0.01.


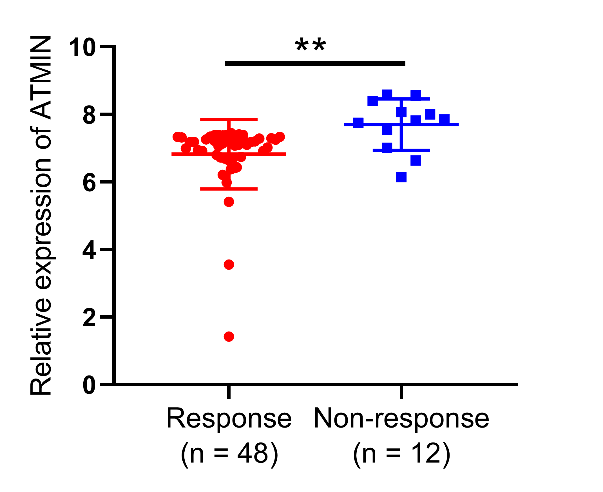


**Supplementary Fig. S2.** The correlation between the mRNA expression levels of ATMIN and LCK, CYP11A1, CYTH4, NAV3, PSTPIP1, EIF4EBP2, FKBP1A or DUSP2, KALRN, or SSUH2 genes in 113 NPC patients from the GSE102349 dataset. The strength of the relationship was evaluated using Pearson’s correlation analysis.


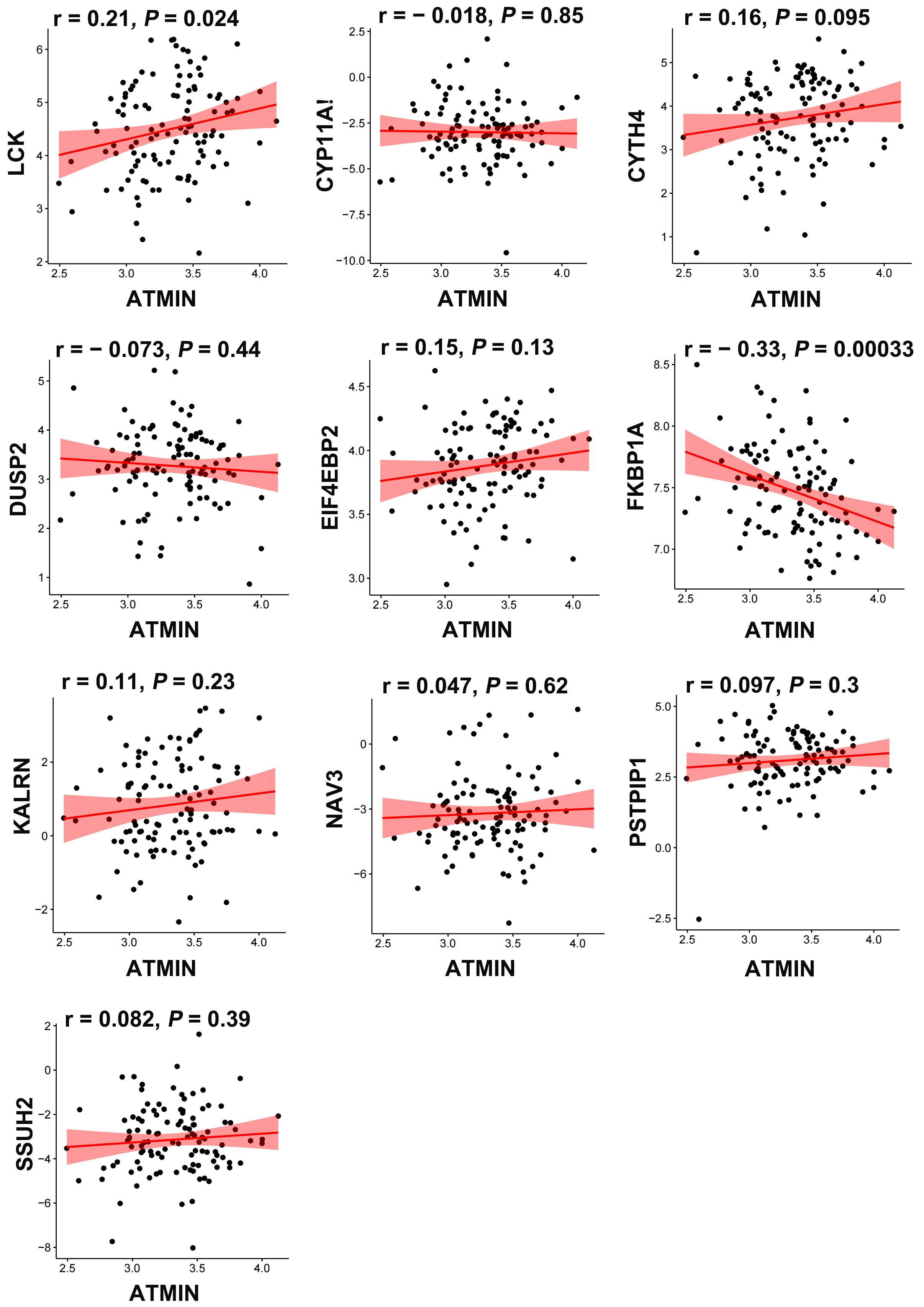

Supplement: Supplementary file 1 — Supplementary material [file 41419_2024_6496_MOESM1_ESM.docx]
