# Supplementary material for: Transcription factor ATMIN facilitates chemoresistance in nasopharyngeal carcinoma
Source: Cell Death Dis. 2024 Feb 6;15(2):112. doi: 10.1038/s41419-024-06496-x (PMC10847093; doi:10.1038/s41419-024-06496-x)

**Supplementary Fig. S3.** The unprocessed immunoblot bands for the indicated figure bands.


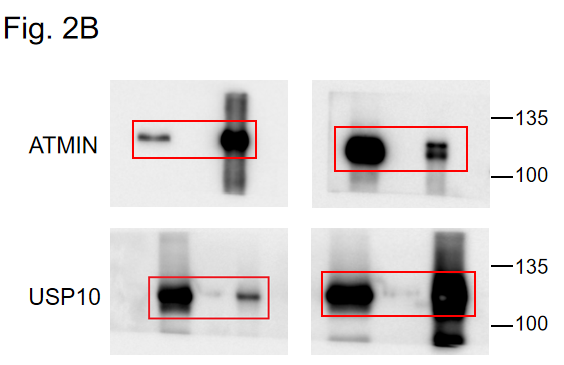

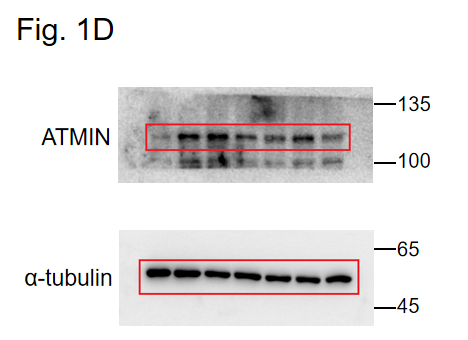


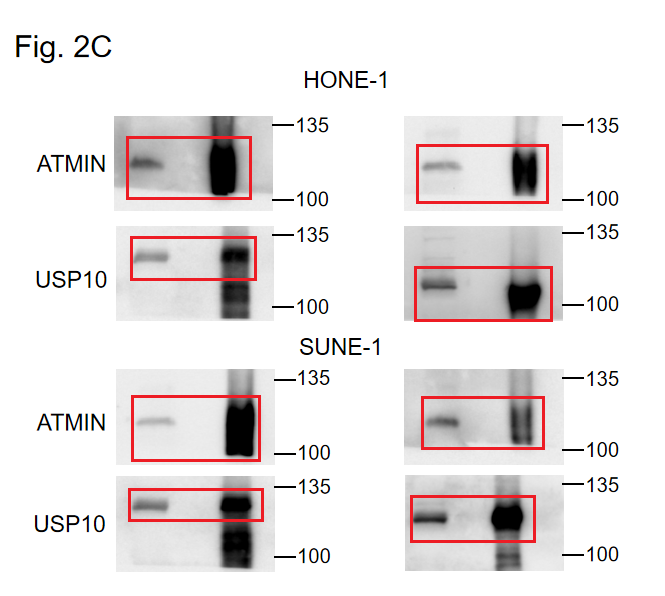


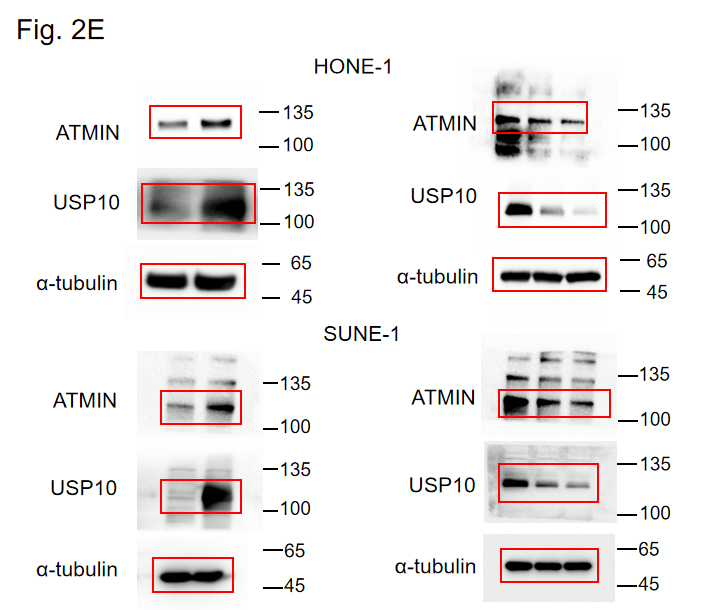


**Supplementary Fig. S3 (cont’d).** The unprocessed immunoblot bands for the indicated figure bands.


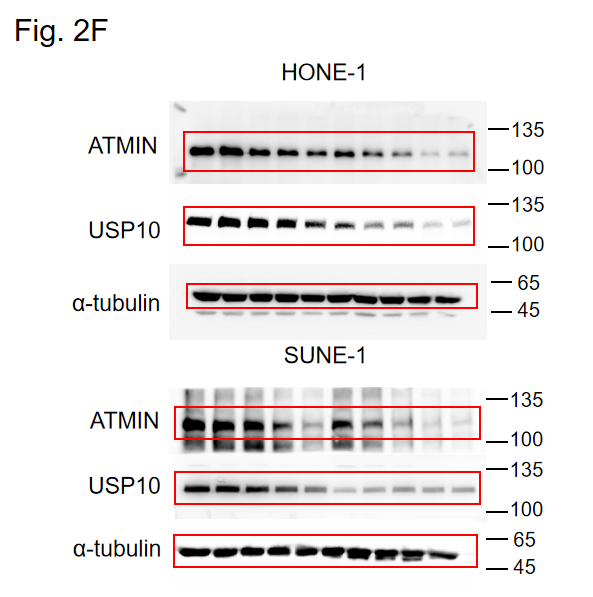


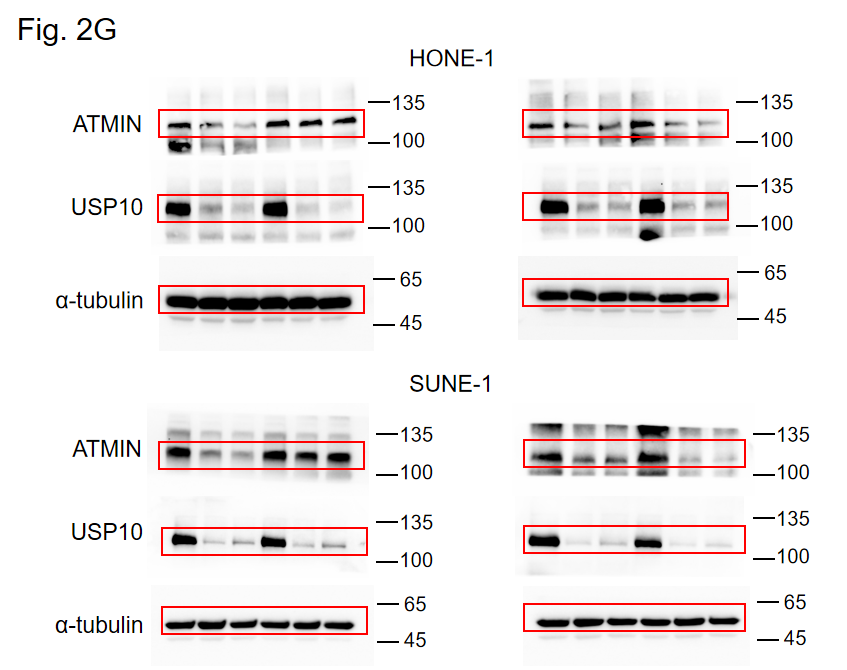


**Supplementary Fig. S3 (cont’d).** The unprocessed immunoblot bands for the indicated figure bands.


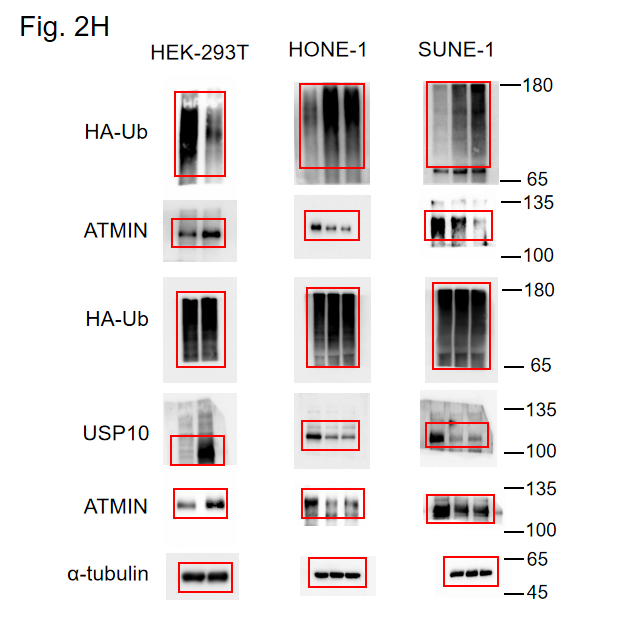


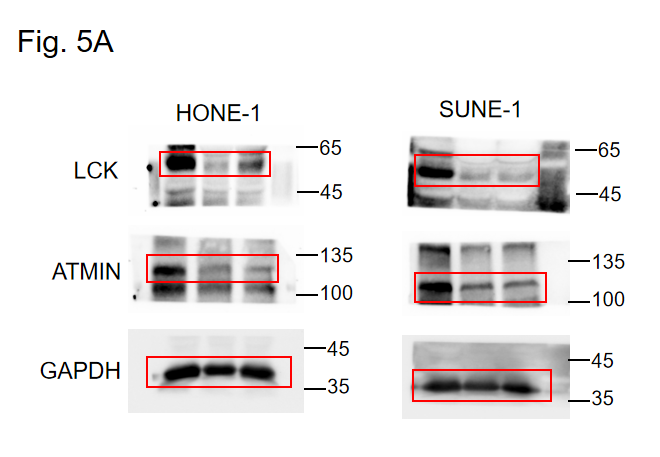

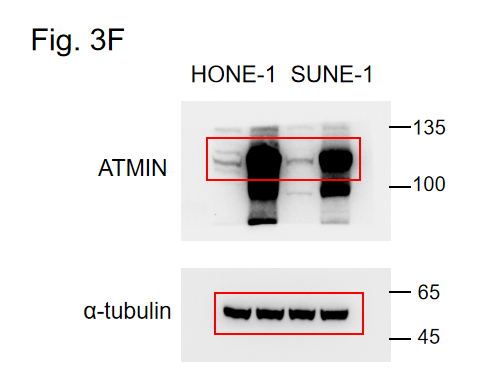

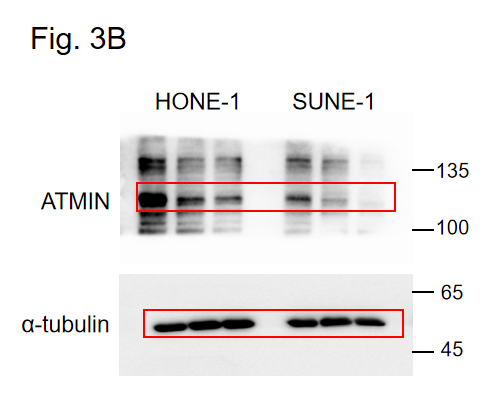

Supplement: Supplementary file 2 — Original western blots [file 41419_2024_6496_MOESM2_ESM.docx]
